# Impact of Educational Activity Formats, Online or In-Person, on the Intention of Medical Specialists to Adopt a Clinical Behaviour: A Comparative Study

**DOI:** 10.1080/28338073.2024.2363550

**Published:** 2024-06-12

**Authors:** Gloria Ayivi-Vinz, Martin Tremblay, Souleymane Gadio, Suélène Georgina Dofara, Sam J Daniel, Denis Talbot, France Légaré

**Affiliations:** a Faculty of Medicine, Université Laval, Quebec, Canada; b Faculty of Science and Engineering, Université Laval, Quebec, Canada; c Canada Research Chair in Shared Decision Making and Knowledge Translation, Université Laval, Quebec, Canada; d VITAM - Centre de recherche en santé durable, CIUSSS de la Capitale-Nationale, Quebec, Canada; e Direction du Développement Professionnel Continu, Fédération des Médecins Spécialistes du Québec, Montreal, QC, Canada; f Centre de Recherche du Centre Hospitalier, Universitaire de Québec-Université Laval, Québec, Canada

**Keywords:** Online learning, in-person learning, distance education, continuing medical education, continuing professional development

## Abstract

COVID-19 accelerated continuing professional development (CPD) delivered online. We aimed to compare the impact of in-person versus online CPD courses on medical specialists’ behavioural intentions and subsequent behaviour. In this comparative before-and-after study, medical specialists attended in-person courses on nine clinical topics. A second group attended an adapted online version of these courses. Behavioural intention and its psychosocial determinants were measured before and immediately after the courses. Behaviour change was measured six months later. Generalised estimating equation (GEE) models were used to compare the impact of course formats. A total of 82/206 in-person registrants (mean age: 52±10 years; 50% men) and 318/506 on-line registrants (mean age: 49±12 years; men: 63%) participated. Mean intention before in-person courses was 5.99±1.31 and 6.43±0.80 afterwards (average intention gain 0.44, CI: 0.16–0.74; *p*=0.003); mean intention before online courses was 5.53±1.62 and 5.98±1.40 afterwards (average intention gain of 0.45, CI: 0.30–0.58; *p*<0.0001). Difference in intention gain between groups was not statistically significant. Behaviour reported six months later was not significantly associated with post-course intention in either group. However, the intention difference increased significantly among those who said they had adopted the targeted behaviour (paired wilcoxon test: *n* = 40 and *p*-value=0.002) while it did not increase significantly in the group of those who had not adopted a targeted behaviour (paired wilcoxon test: *n* = 16 and *p*-value=0.223).

In conclusion, the increase in intention of specialists after CPD courses was similar whether the course was in-person or online. Also, an increase in intention in both groups signalled more likelihood of adoption.

## Introduction

Continuing professional development (CPD) courses help healthcare professionals maintain and improve their knowledge and skills as well as apply them in their practices [1–3]. Physicians are some of the most frequent users of CPD courses [4]. CPD developers use a variety of course formats, including online learning, in-person [5], or blended formats [6–9]. During the COVID-19 pandemic, virtual learning formats predominated, the offer of online medical education activities increased, and engagement in online continuing medical education by physicians and other healthcare professionals rose. Almost half of accredited organisations delivered activities addressing pandemic-related topics, mostly in online formats [10]. CPD developers adopted innovative design approaches to capitalise on the affordability of education using digital technologies and to increase engagement in and accessibility of their courses [11]. Some studies suggest that where face-to-face health professional training is not possible, online learning activities provide an effective alternative in terms of producing comparable student test performance – but only for high academic performers. For lower performing students, online courses produced significantly poorer student test results than in a traditional face-to-face environment [12,13]. Several systematic reviews and meta-analyses [5,9,14,15] reveal that the impact of CPD courses varies according to the course formats (online, in-person, or blended), although the level of evidence is low. However, most evidence on the effectiveness of online compared to in-person courses focuses on the lowest level of Kirkpatrick’s learning levels, i.e. knowledge acquisition [16]. Consequently, there is a lack of data about whether this newly acquired knowledge translates into clinical behaviour [17].

Socio-cognitive theories of individuals’ behaviour change are well-suited for supporting the design of CPD courses for health professionals and for assessing their impact on participants’ clinical behaviour. In particular, the integrated theory of behaviour change in health professionals [18] identifies the modifiable psychosocial factors that influence change in clinical behaviour or medical practice. This theory assumes that behavioural intention is a proxy for health professionals’ behaviour. While some studies have observed a predictable relationship between health professionals’ intentions and their subsequent behaviour, others question intention as a valid proxy, and few studies have followed up to see if behaviour change did in fact occur [15,19–21]. Therefore, we aimed to compare the impact of the CPD course format, in-person or online, on the behavioural intention of medical specialists in Quebec, Canada, and to follow up six months later to see if the intention had translated into clinical behaviour.

## Materials and Methods

### Study Design

This study is a comparative study using two datasets (one in 2019 and another from 2020 to 2022) with pre- and post-intervention measures. The results are presented following the recommendations of the “Strengthening the Reporting of Observational Studies in Epidemiology” (STROBE) guide [22].

### Setting and Interventions

The Fédération des Médecins Spécialistes du Québec (FMSQ) includes thirty-five medical associations with around 10,000 physician members in 59 specialties [23]. The Interprofessional Training Day is an annual meeting where the FMSQ provides CPD courses to keep abreast of best medical practices [24]. This study analysed datasets from two quasi-experimental studies and thus two intervention groups (A and B). Intervention A was attendance at an in-person CPD course at the Training Day in 2019 in Quebec City. The CPD courses were accredited by the FMSQ’s CPD department, which is an authorised CPD provider of the Royal College of Physicians and Surgeons of Canada (Sections 1 and 3) [1]. Section 1 accredited courses are group learning activities (e.g. conferences), and Section 3 includes self-assessment programmes and activities. These courses covered a range of objectives and competencies covering nine topics (Appendix 2). According to the principle of “target-action-context-time” [25], the FMSQ defines a specific target behaviour for each course. Intervention A courses included didactic lectures, interactive activities, and discussion workshops. Intervention B consisted of watching a series of short video recordings of the same courses combined with quizzes and was available in an asynchronous online learning format. The online courses were accessible on the FMSQ’s learning management system, MÉDUSE. This study analysed the FMSQ databases from the training day 2019 (in-person courses) and from the MÉDUSE platform (online courses) from 2020 to 2022.

There were three data collection periods for each intervention: before the CPD courses (T0), after the CPD courses (T1), and follow-up at six months (T2). At T0, participants completed a socio-demographic questionnaire and the CPD-REACTION questionnaire (Appendix 1), a validated tool [26,27] that measures behavioural intention and the psychosocial determinants of intention. Participants also completed the CPD-REACTION questionnaire immediately after attending a course (T1). Participants who attended more than one CPD course completed the CPD REACTION questionnaire at T0 and T1 for each course attended. As for follow-up, six months after the course (T2), participants were invited to complete a new questionnaire measuring self-reported behaviour (Appendix 3). Using open-ended questions, this questionnaire collected information on whether the participant had adopted the behaviour targeted by the course and on the barriers and facilitators to their adoption of the behaviour.

### Participants

The target population was medical specialists in the province of Quebec, Canada. Eligible participants were medical specialists who had completed at least one CPD course organised by the FMSQ. We included physicians who participated in at least one of the CPD activities at the 2019 Interprofessional Training Day in person or in at least one of the same online learning CPD activities on the MÉDUSE platform between 2019 and 2022. We excluded physicians who did not consent to participate or did not consent to complete the CPD-REACTION questionnaires both before and after the course.

### Variables and Data Sources

The dependent variable of the study was the behavioural intention of CPD course attendees to adopt a behaviour. It was assessed using items “I intend to [adopt targeted behaviour] … ” and “I plan to [adopt targeted behaviour] … ” in the CPD-REACTION questionnaire (Appendix 2). Behavioural intention is a continuous quantitative variable with values ranging from 1 (low intention) to 7 (high intention). Independent variables were sociodemographic characteristics and psychosocial determinants of intention, i.e. four modifiable factors that influence behavioural intention, also measured using the CPD-REACTION questionnaire. These psychosocial variables were social influence, beliefs about abilities, beliefs about consequences, and moral norms. The score for each psychosocial variable was obtained by calculating the mean scores for the items relating to that variable. Each variable is measured using 2 to 3 items ranging from 1 to 7. Data collected on participants’ socio-demographic characteristics were gender, age, and medical affiliation. The latter was extracted from the participant’s affiliated medical associations and recoded according to the main specialty areas covered by the FMSQ. Data collected on course characteristics included the topics and format of the courses. The course topics were patient safety, healthcare accidents, optimising care, intraoperative pain management and opioids, sports injuries or musculoskeletal conditions, eating disorders, attention deficit and hyperactivity disorder, cardio-oncology, and local anaesthesia. The course formats were either in-person or online.

### Statistical Analysis

Statistical analyses were performed using SAS version 9.4. As study participants attended one or more courses, with a before and after measurement for each course attended, there was a dependency between observations from the same individual. In addition, intention score values ranged from 1 to 7. Therefore, generalised estimating equation (GEE) models were used as they do not require specific assumptions on the error distribution or correlation structure [28–31]. The PROC GENMOD procedure was used, with a normal working distribution, an identity link function, and an exchangeable working correlation matrix. The GEE model included terms for the course format, terms for time (T0 vs T1), and an interaction between group and time, and was adjusted for participants’ socio-demographic variables such as age, gender, and specialty area. The results are presented as differences in means (DM) between T0 and T1, with their 95% confidence intervals, for the course format and each of the 9 CPD course topics. We then included an interaction between course format and course topics to check whether the effect of the course format varied according to the course topics. This model was adjusted for course characteristics (format and content) and participants’ socio-demographic variables such as age, sex, and specialty area. Finally, the regression model’s assumptions were verified, and appropriate corrections were incorporated into the final model [30,32]. We also investigated the factors influencing post-course behavioural intention in a GEE regression of intention at T1 according to the psychosocial variables at T0. We used a Fisher exact test to compare the proportion of behaviour adoption according to the course format. Using a Wilcoxon test, we examined the association between intention after courses and behaviour adoption at the six-month follow-up, as well as the association between intention difference and behaviour adoption.

## Results

### Flow and Characteristics of the Study Population

**Figure 1.**
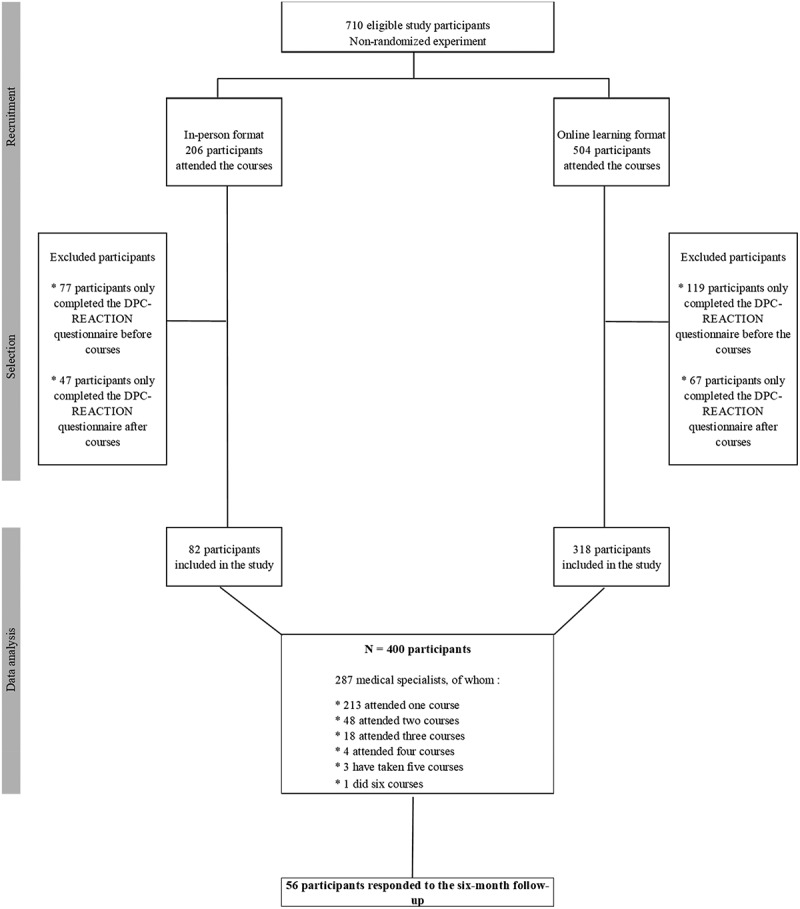
Flow chart of study participants.

During data collection, a total of 710 participants − 206 in-person and 504 online – attended the CPD course courses produced by the FMSQ. A total of 400 participants met the inclusion criteria: 82 participants for the in-person courses and 318 for the online courses (Figure 1). As for the number of courses attended in the in-person format, 96% or 76 physicians participated in a single course, and 4% or three physicians attended two courses. For the online format, 68%, or 144 physicians, participated in a single course, and 30% or 68 physicians attended two to six courses. In addition, four physicians participated in some of the in-person courses and some of the online courses. Consequently, the 400 total participants in the various courses corresponded to 287 medical specialists included in the study (Figure 1).

The average age of the participants was 50 ± 12 years. Among the 400 study participants, most were men (60% or 240). The medical specialties most represented were surgery, clinical, and laboratory (64%, 23%, and 12%, respectively) (Table 1). Furthermore, the socio-demographic profiles of participants in each course format seemed different in terms of age, sex, and medical field. There were as many men as women for the in-person format, while for the online format, there were more men. More participants in the in-person format were working in clinical fields (vs. in surgery or labs) than in the online format. In-person participants were older than online participants.

**Table 1.** Socio-demographic profile of participants.

	All participants	In-person format	Online learning format
Variables	N = 400	N = 82	N = 318
Age (years), mean ± SD	50 ± 12	52 ± 10	49 ± 12
Sex, n (%)			
Male	240 (60)	41 (50)	199 (63)
Female	160 (40)	41 (50)	119 (37)
Profession, n (%)			
Specialist	396 (99)	82 (100)	314 (99)
Other	4 (1)	0 (0)	4 (1)
Clinical domain, n (%)			
Surgery	257 (64)	39 (47)	218 (68)
Clinic	91 (23)	31 (38)	60 (19)
Laboratory	46 (12)	12 (15)	34 (11)
Other	6 (1)	0 (0)	6 (2)

### Comparison of Behavioural Intention by Course Format

Before the courses (T0), the average behavioural intention was 6.0 ± 1.3 for the in-person format and 5.5 ± 1.6 for the online format. After the courses (T1), the average behavioural intention was 6.4 ± 0.80 for in- person courses and 6.0 ± 1.4 for online courses (Table 2). At baseline (T0), the average participants’ behavioural intention differed between in-person and online formats (p-value <0.001). The comparative gains observed for the in-person and online formats were not statistically significantly different (interaction time*format p-value = 0.80). Table 3 shows the mean difference in intention before and after courses according to format.

**Table 2.** Dispersion parameters for physicians’ behavioural intention.

	n	Mean ± SD	Median	Interquartile range	Min	Max
In-Person Format						
T0-Before	81	6.0 ± 1.2	6.5	5.0–7.0	1	7
T1-After	82	6.4 ± 0.8	7.0	6.0–7.0	3	7
Online Format						
T0-Before	315	5.5 ± 1.5	6.0	5.0–7.0	1	7
T1-After	316	6.0 ± 1.1	6.0	5.8–7.0	1	7

**Table 3.** Impact of course on medical specialists’ intention.

		Intention		
	N	Means difference (SE^*^)	CI 95%	P-value
Unadjusted model for all participants				
*After vs. Before*	397	0.47 (0.09)	[0.30; 0.64]	<0.0001
Adjusted** model for all participants				
*After vs. Before*	397	0.47 (0.09)	[0.30; 0.65]	<0.0001
Adjusted** model for in-person format				
*After vs. Before*	81	0.45 (0.15)	[0.16; 0.74]	0.0029
Adjusted model** for online format				
*After vs. Before*	316	0.44 (0.07)	[0.30; 0.58]	<0.0001

*standard error; **adjustment variables: topics, age, sex, and medical field.

## Psychosocial Factors of Intention per Course Format and Association with Behavioural Intention

**Figure 2.**
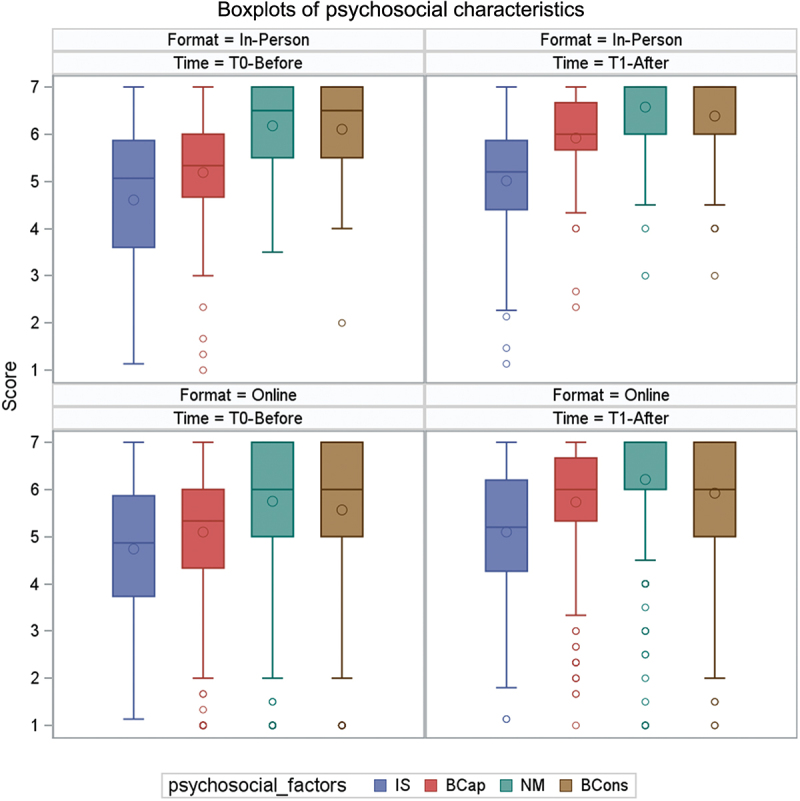
Box plots of psychosocial factors of intention.

Notes: IS refers to Social Influence; BCap refers to Beliefs about Capabilities; NM refers to Moral Norms; BCon refers to Beliefs about consequences

Before CPD courses, median scores for social influence were 5.1 (interquartile range: 3.6–5.9) for in-person courses and 4.9 (interquartile range: 3.7–5.9) for online courses. As for beliefs about capabilities, median scores were 5.3 (interquartile range: 4.7–6.0) for in-person courses and 5.3 (interquartile range: 4.3–6.0) for online courses. The median scores for moral norms and beliefs about consequences were 6.5 (interquartile range: 5.5–7.0) for in-person courses and 6.0 (interquartile range: 5.0–7.0) for online courses. Figure 2. illustrates distributions of the psychosocial factors of intention. The mean gain at T1 in courses in both formats was statistically significant for all psychosocial factors (Table 4).

**Table 4.** Changes in psychosocial factors before and after courses.

	T0-Before			T1-After			Difference	
	n	Mean	SD	n	Mean	SD	Mean	CI 95%
**In-person format**								
Social influence	82	4.6	1.5	82	5.0	1.3	0.41	[0.31;0.50]
Beliefs about capabilities	80	5.2	1.3	82	5.9	1.0	0.73	[0.63; 0.83]
Moral standards	82	6.2	0.9	81	6.6	0.8	0.40	[0.33; 0.47]
Beliefs about consequences	82	6.1	1.1	82	6.4	0.9	0.28	[0.20; 0.36]
**Online Format**								
Social influence	318	4.7	1.4	318	5.1	1.3	0.36	[0.33; 0.39]
Beliefs about capabilities	315	5.1	1.5	318	5.7	1.2	0.64	[0.60; 0.67]
Moral standards	313	5.8	1.5	315	6.2	1.2	0.46	[0.41; 0.51]
Beliefs about consequences	318	5.6	1.4	318	5.9	1.2	0.36	[0.32; 0.39]

SD: Standard Deviation, CI: Confidence Interval.

After CPD courses, beliefs about capabilities, beliefs about consequences, and moral norms were the factors associated with behavioural intention. For fixed scores of beliefs about consequences and moral norms, an increase in beliefs about capabilities was associated with a 0.16 rise in behavioural intention score (CI [0.03; 0.28], p-value = 0.016). Moreover, increased beliefs about consequences were associated with a 0.29 rise in behavioural intention score (CI [0.14; 0.45], p-value = 0.0003) for fixed scores of beliefs about capabilities and moral norms. Finally, an increase in moral norms was associated with a 0.20 rise in behavioural intention score (CI [0.009; 0.399], p-value = 0.04) for fixed scores of beliefs about capabilities and consequences.

## Follow Up from Intention to Behaviour

Among 400 participants, 56 responded to the follow-up questionnaire at six months (T2): 36 had taken the in-person courses, and 20 attended the online courses. Overall, 16 participants (29%) reported not adopting the targeted behaviour. Of these, 31% (*n* = 31) were in-person attendees and 25% (*n* = 5) were online attendees. The remaining 71% (*n* = 40) said they had adopted at least one of the target behaviours; of these 69% (*n* = 25) were in-person attendees and 75% (*n* = 15) were online attendees. Six months after the course, the proportion of behaviour change reported was similar between in-person and online attendees (Fisher exact test: *n* = 56 and p-value = 0.76). Table 5 shows self-reported behaviour adoption according to the course format.

**Table 5.** Frequencies of self-reported behaviour according to CPD course format (*N* = 56).

	Behaviour change reported		Total
	No	Yes	
Format, n (%)			
In-Person	11 (31)	25 (69)	36 (64)
Online	5 (25)	15 (75)	20 (36)
Total	16 (29)	40 (71)	56 (100)

**Figure 3.**
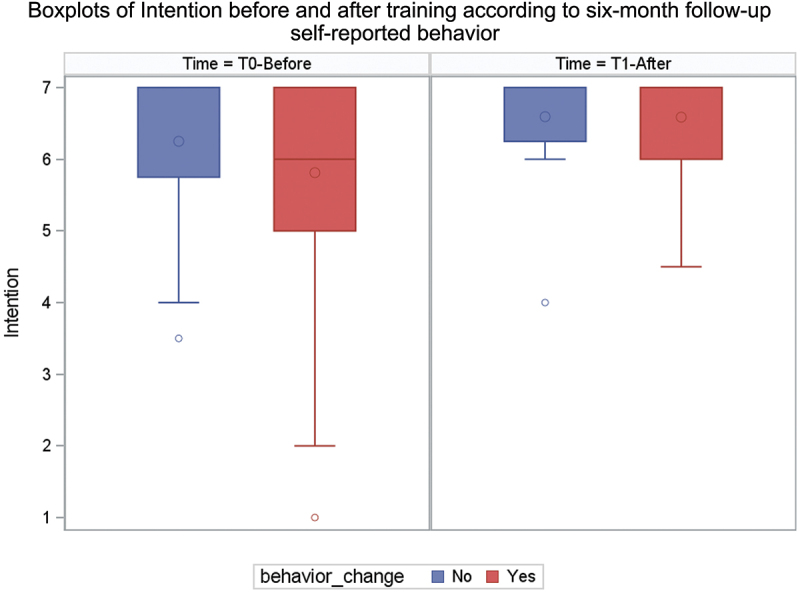
Box plots of intention after course according to self-reported adoption or not of target behaviour at six-month follow-up.

**Figure 4.**
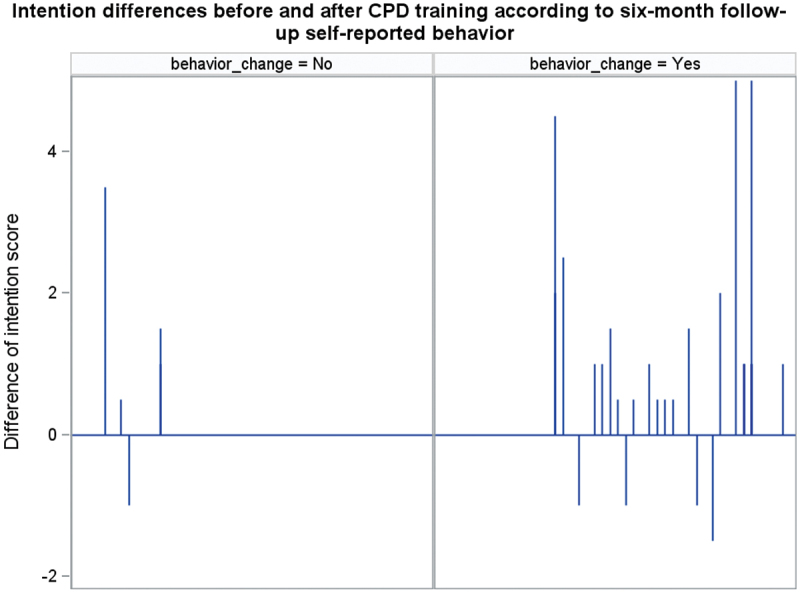
Observed gain or loss of intention compared to self-reported behaviour six months after CPD course.

The behaviour reported six months after CPD courses (T2) was not significantly associated with the intention immediately after the course in either group (T1) (Wilcoxon test: *n* = 56 and p-value = 0.49) (Table 6). Figure 3 shows a ceiling effect in behavioural intention for both those who did and did not adopt the target behaviours. However, Figure 4 highlights that for participants who did not adopt the target behaviour, the difference in intention had values closer to zero than for participants who did adopt it. The majority of participants who had adopted the targeted behaviour also showed a gain in intention after the course. Table 7 shows that the intention difference increased significantly among those who said they had adopted the targeted behaviour (paired Wilcoxon test: *n* = 40 and p-value = 0.002). The intention difference did not increase significantly in the group of those who had not adopted a targeted behaviour (paired Wilcoxon test: *n* = 16 and p-value = 0.223).

**Table 6.** Association between intention and self-reported behaviour (*N* = 56).

	n	Mean ± SD	P-value of test of Wilcoxon
Intention T1-after course			
Targeted behaviour adopted	40	6.59 ± 0.7	0.42
Targeted behaviour not adopted	16	6.59 ± 0.8

**Table 7.** Self-reported behaviour and association with behavioural intention’s evolution before-after courses (*N* = 56).

Follow-up after six months	n	Difference of intention T1 -T0 mean ± SD	P-value of paired Wilcoxon test
Targeted behaviour adopted	40	0.78 ± 1.4	0.002
Targeted behaviour not adopted	16	0.34 ± 1.0	0.223

## Discussion

We compared the impact of the CPD course format, in-person or online, on the behavioural intention of medical specialists in Quebec and followed up on the adopted behaviours six months later. We found that medical specialists’ behavioural intention increased significantly after all CPD courses, with no significant difference between in-person and online formats in this gain of intention. At the follow-up, the proportion of behaviour change reported was not significantly different between in-person and online participants. Furthermore, we did not notice a significant association between intention assessed after the courses and self-reported behaviour at six months. However, a gain in intention was observed amongst those who reported a behaviour change six months later. Our findings lead us to make the following observations.

First, the results suggest that CPD courses of both kinds increased the behavioural intention of medical specialists. This result is consistent with the published evidence that shows CPD activities improve practitioners’ knowledge and behaviour [33]. The CPD courses provided to medical specialists were mainly associated with increased knowledge and intention to practice the acquired skills. In contrast, Wallace and collaborators highlighted that CPD activities such as conferences, lectures, and symposiums, have been found to have a limited effect on improving practitioner competence and performance and no significant effect on patient health outcomes [34]. Further studies should explore the association between knowledge, intention (as a proxy for behaviour change), and health outcomes, i.e. the three highest levels of the Kirkpatrick framework.

Second, in our study, there was no significant difference in the increase of intention before and after CPD courses according to the CPD course format. Moreover, when we conducted a sub-analysis on the musculoskeletal conditions and local anaesthesia courses only, as these were most frequent topics in both online and in-person courses, course format still did not impact intention gain. These results confirm the findings of certain studies showing that the online format is generally at least as effective as the in-person format [9,35]. However, while Cervero et al. concluded that there is no consensus in the literature that e-learning has a greater effectiveness than in-person learning [9], a meta-analysis of 93 studies suggested that blended learning formats were more effective than exclusively online or in-person learning regarding the level of knowledge acquisition [14]. This evidence is coherent with our findings that highlighted, for the same topics, a similar association of CPD courses with behavioural intention, regardless of course format. During the pandemic, various in-person course programmes were adapted to online formats to facilitate distance learning and make them accessible during lockdown. In light of our findings, we suggest that when course materials are produced for the same learning objective, the impact of the CPD course remains similar for in-person and online formats. Post-pandemic, however, it could be relevant to combine in-person and online formats for CPD activities [14,36]. In a study published in 2020, Vallée et al. reviewed 56 studies to evaluate several types of learning support in a mixed format [36]. This review compared blended learning (in-person and online activities) with in-person learning in health education, and showed that knowledge acquisition was higher with the combined learning format [36]. Lockey and collaborators also demonstrated superior knowledge acquisition for courses in blended format compared to in-person format [14].

Third, we found no association between behavioural intention after the courses and self-reported behaviour at six months. However, a gain in intention was observed amongst those who reported a behaviour change six months later. Some studies have addressed the comparative effectiveness of online versus in-person courses concerning knowledge acquisition [9,14,35]. In contrast, few studies have addressed the comparative effectiveness of online versus in-person courses concerning Kirkpatrick’s Level III and IV indicators [37,38] (behaviour change and impact on patient outcomes). Indeed, most CPD activities do not target clinical behaviour change at all [39]. Our study addressed Kirkpatrick’s level III by checking intention and self-reported behaviour after six months. Adopting targeted behaviour at six months was not associated with intention immediately after courses. These results could be due to the ceiling effect, as the score ranges from 1 to 7, and the higher values are both near 7. As the association between intention after courses and behaviour adoption seemed biased due to the ceiling effect [40], we checked if there was a difference in intention before and after courses, stratified by reported behaviour change (yes or no) and found a significant intention gain among behaviour adopters. This result supports the evidence suggesting that behavioural intention is a relevant proxy for behaviour.

The present study has limitations. Foremost, not using a probability sampling method limited the external validity of the results. Furthermore, the non-response and loss-to-follow-up [41] may have introduced a selection bias that could overestimate the effect of the course on behavioural intention; although this would be the case in both the in-person and online course formats. In particular, analyses regarding behaviour adoption at six months were conducted on a limited sample and should be interpreted cautiously. Second, we suspected incontrollable confounding bias since the year of CPD activities and course format are confounded by course content. Third, there could be information bias due to the timing of the intervention: the in-person courses were carried out before the pandemic, and the online courses were carried out during the pandemic. The slowdown in specialist physicians’ activities and confinement could have led to physicians systematically underestimating their behavioural intention to adopt targeted behaviours not directly related to the management of COVID-19. Fourth, while in-person courses included interactive discussion opportunities, the online versions included short pre-recorded videos and quizzes but no further interactive elements. Had both courses had interactive elements, our comparison may have been more meaningful. Some authors have found that lack of interactivity is barrier to online learning [42–44]. However, in 2019, a systematic review and meta-analysis on effectiveness of digital education for medical students found no difference in post intervention skills between more and less interactive forms of digital education [45].

Finally, a study design such as a randomised controlled trial or a non-inferiority trial could better estimate a direct causal effect of format on behavioural intention and adoption. In terms of strengths and prospects, this study established evidence in a real-life context with data provided by the Fédération des Médecins Spécialistes du Québec. Also, further studies could examine differences in intention between in-person and online courses whose formats had similar levels of interactivity.

## Conclusion

Although the behavioural intention of Quebec medical specialists increased following CPD courses, the format of the course, whether in-person or online, did not appear to have a distinct impact. The results of this study provide helpful evidence for CPD program developers on learning formats that facilitate access to CPD courses and the adoption of targeted clinical behaviours. In the short term, our findings on the lack of significant differences in behavioural intention according to course format will help CPD course organisers (including the FMSQ) improve the effectiveness of their courses. In the medium term, this study contributes to enhancing knowledge of the effects of online and in-person CPD on behavioural intention. In the long term, this study will contribute to improved patient health, better use of healthcare resources, and an improved healthcare system in Quebec.

## Appendix 1:

### Appendices Appendix 1: CPD-REACTION Questionnaire and Scoring Procedure

**Table:**

Construct scale		Item^a^	Response choice	Pre‐coded item value^b^	Final item score^c^	Score by construct^d^
Intention	I_1_	I intend to [*behavior*]	Strongly disagree/agree	1 to 7	1 to 7	(I_1_+I_7_)/2
	I_7_	I plan to [*behavior*]	Strongly disagree/agree	1 to 7	1 to 7	
Social influence	I_2_	To the best of my knowledge, the percentage of my colleagues who [*behavior*] is…	0‐20% 21‐40% 41‐60% 61‐80% 81‐100%	1 2 3 4 5	1.4 2.8 4.2 5.6 7	(I_2_+I_6_+I_9_)/3
	I_6_	Now think about a co‐worker whom you respect as a professional. In your opinion, does he/she [*behavior*]?	Never/Always	1 to 7	1 to 7	
						
	I_9_	Most people who are important to me in my profession [*behavior*]	Strongly disagree/agree	1 to 7	1 to 7	
						
Beliefs about capabilities	I_3_	I am confident that I could [*behavior*] if I wanted to.	Strongly disagree/agree	1 to 7	1 to 7	(I_3_+I_5_+I_11_)/3
	I_5_	For me, [*behavior*] would be…	Extremely difficult/easy	1 to 7	1 to 7	
	I_11_	I have the ability to [*behavior*]	Strongly disagree/agree	1 to 7	1 to 7	
Moral norm	I_4_	[*Behavior*] is the ethical thing to do.	Strongly disagree/agree	1 to 7	1 to 7	(I_4_+I_10_)/2
	I_10_	It is acceptable to [*behavior*]	Strongly disagree/agree	1 to 7	1 to 7	
Beliefs about consequences	I_8_	Overall, I think that for me [*behavior*] would be…	Useless/Useful	1 to 7	1 to 7	(I_8_+I_12_)/2
	I_12_	Overall, I think that for me [*behavior*] would be…	Harmful/Beneficial	1 to 7	1 to 7	

^a^Number item (e.g. I1= Item 1); ^b^ Pre‐coded item value is an assigned value on a Likert scale (e.g. Strongly disagree = 1, Strongly agree = 7; never = 1, always = 7, etc.); ^c^ Final item score is the score by item for each participant (possible scale range = 1 to 7).

^b^Score by construct = mean score by construct (possible scale range = 1 to 7). Note: for constructs with two items, no imputed values are possible. For constructs with three items, the raw score of the scale is missing if two or more items are missing. In the case of one missing, the missing item is imputed by the mean of the two others items.

## Appendix 2:

### Appendix 2: Overview of courses

**Table:**

CPD Course	Main behavior	In-person format					Online format				
		Duration	Number of lecturer	Group work and experience sharing	Quiz	Discussions and questions	Duration	Number of lecturer	Group work and experience sharing	Quiz	Discussions and questions
1 Essentials of Patient Safety Education and Assessment (Patient Safety)	Teach my learners the principles of patient safety and continuous improvement in the hospital setting	4h	3	Yes	No	interactive	4h45	3	No	Yes	NA
2 A care incident is more than just a mishap! (Care incident)	Complete an incident/accident report according to the guidelines in effect in my institution when a care incident occurs	4h	4	Yes	No	interactive	3h30	4	No	Yes	NA
3 The ERC initiative: an innovative interdisciplinary approach to optimizing care (Optimizing care)	Use at least one of the 6 recommendations of Optimized Recovery Canada when managing perioperative patients	4h	2	No	No	interactive	1h30	2	No	Yes	NA
4 Can the opioid crisis be contained through optimal perioperative pain management? (Perioperative pain-Opioid)	Prescribe opioids in a safe manner when managing perioperative pain in my patients	4h	2	No	Yes	interactive	3h30	2	No	Yes	NA
5 Sports injuries: assessment and treatment of common musculoskeletal conditions (Sports injuries)	Use a clinician-radiologist approach to investigate common musculoskeletal injuries in active patients	8h	13	No	Yes	at the end of the course	9h	13	No	Yes	NA
6 Eating disorders in adolescents (Eating disorders)	Systematically assess the medical and psychological health status of my patients with eating disorders	8h	6	Yes	Yes	interactive	6h30	4	No	Yes	NA
7 ADHD is also a matter of the heart (Attention deficit)	To use psychostimulants in a safe manner for ADHD patients with heart problems	4h	4	No	No	Noe	3h45	4	No	Yes	NA
8 Cardio-oncology, to get to the bottom of it! (Cardio-oncology)	Use a cardiologist-hemato-oncologist approach to manage cardiovascular risks induced by hemato-oncology in my patients	4h	4	No	No	Noe	3h15	3	No	Yes	NA
9 Local anesthesia: when blocking rhymes with toxicity (Local anesthesia)	Follow the 2018 ASRA recommendations for the management of systemic toxicity resulting from local anesthesia (LAST kit)	4h	6	No	No	interactive	3h	6	No	Yes	NA

## Appendix 3: Questionnaire to be Completed 6 Months After the Educational Intervention (Translated from the French Original)

Survey

**Q1**. Please enter the identification code you used at the start of this training activity. This is your year of birth followed by your mother’s initials. For example, if you were born in 1970 and your mother’s name is Anne-Marie Cloutier, your code would be 1970AMC. [open-ended text]

**Q2**. As a consequence of taking the course “Essentials of patient safety education and evaluation,” have you taught trainees about the principles of patient safety and continuous improvement in hospitals?

- Yes
- No
- I did not attend this JFI 2019 course.

**Q3**. As a consequence of taking the course “A care accident, much more than an ordinary accident,” have you produced an incident or accident report in accordance with the guidelines of your institution when a care accident occurred?

- Yes
- No
- I did not attend this JFI 2019 course.

**Q4**. As a consequence of taking the course “The ORC initiative: an innovative interdisciplinary approach to optimizing care,” have you applied at least one of the 6 recommendations of Optimized Recovery Canada in the perioperative management of patients?

- Yes
- No
- I did not attend this JFI 2019 course.

**Q5**. As a consequence of taking the course “Can the opioid crisis be contained through best practices in perioperative pain management?” have you prescribed opioids for pain in a safe way?

- Yes
- No
- I did not attend this JFI 2019 course.

**Q6**. As a consequence of taking the course “Sports injuries: assessment and treatment of common musculoskeletal conditions,” have you used a clinician-radiologist approach to the investigation of common musculoskeletal injuries in active patients?

- Yes
- No
- I did not attend this JFI 2019 course.

**Q7**. As a consequence of taking the course “Eating disorders in adolescents,” have you systematically assessed the medical and psychological state of the health of your patients with eating disorders?

- Yes
- No
- I did not attend this JFI 2019 training.

**Q8**. As a consequence of taking the course “ADHD is also a heart problem,” have you used psychostimulants in a way that was safe for ADHD patients with heart problems?

- Yes
- No
- I did not attend this JFI 2019 course.

**Q9**. As a consequence of taking the course “Cardio-oncology with a clear concience,” have you used a cardiology-hemato-oncologist approach to managing the cardiovascular risks of hemato-oncology?

- Yes
- No
- I did not attend this JFI 2019 course.

**Q10**. As a consequence of taking the course “Local anaesthesia: when blocking rhymes with toxicity,” are you following the 2018 ASRA recommendations for the management of systemic toxicity resulting from local anesthesia (LAST toolkit)?

- Yes
- No
- I did not attend this JFI 2019 course.

**Q11**. As a result of the training course(s), have you made one or more other changes to your professional practice?

- Yes. Please explain using an example: __________
- No. Please explain why not : __________
- N/A

**Q12**. In your opinion, did your course have an impact on the safety or health of your patients?

- Yes. Please explain using an example: __________
- No. Please explain why not : __________
- N/A

**Q13**. Would you be willing to take part in a short telephone interview with the investigators of this research project to tell us about your experience?

- Yes. Please write your name and email: __________
- No.
